# Identification of Risk Pathways and Functional Modules for Coronary Artery Disease Based on Genome-wide SNP Data

**DOI:** 10.1016/j.gpb.2016.04.008

**Published:** 2016-12-11

**Authors:** Xiang Zhao, Yi-Zhao Luan, Xiaoyu Zuo, Ye-Da Chen, Jiheng Qin, Lv Jin, Yiqing Tan, Meihua Lin, Naizun Zhang, Yan Liang, Shao-Qi Rao

**Affiliations:** 1 Institute for Medical Systems Biology and Department of Medical Statistics and Epidemiology, School of Public Health, Guangdong Medical College, Dongguan 523808, China; 2 School of Life Sciences, Sun Yat-sen University, Guangzhou 510080, China; 3 Department of Medical Statistics and Epidemiology, School of Public Health, Sun Yat-sen University, Guangzhou 510080, China; 4 Maoming People's Hospital, Maoming 525000, China

**Keywords:** Coronary artery disease, Genome-wide SNP profiling, Risk pathway, Functional module, Genetic network

## Abstract

**Coronary artery disease** (CAD) is a complex human disease, involving multiple genes and their nonlinear interactions, which often act in a modular fashion. Genome-wide single nucleotide polymorphism (SNP) profiling provides an effective technique to unravel these underlying genetic interplays or their functional involvements for CAD. This study aimed to identify the susceptible pathways and modules for CAD based on SNP omics. First, the Wellcome Trust Case Control Consortium (WTCCC) SNP datasets of CAD and control samples were used to assess the joint effect of multiple genetic variants at the pathway level, using logistic kernel machine regression model. Then, an expanded **genetic network** was constructed by integrating statistical gene–gene interactions involved in these susceptible pathways with their protein–protein interaction (PPI) knowledge. Finally, risk **functional modules** were identified by decomposition of the network. Of 276 KEGG pathways analyzed, 6 pathways were found to have a significant effect on CAD. Other than glycerolipid metabolism, glycosaminoglycan biosynthesis, and cardiac muscle contraction pathways, three pathways related to other diseases were also revealed, including Alzheimer’s disease, non-alcoholic fatty liver disease, and Huntington’s disease. A genetic epistatic network of 95 genes was further constructed using the abovementioned integrative approach. Of 10 functional modules derived from the network, 6 have been annotated to phospholipase C activity and cell adhesion molecule binding, which also have known functional involvement in Alzheimer’s disease. These findings indicate an overlap of the underlying molecular mechanisms between CAD and Alzheimer’s disease, thus providing new insights into the molecular basis for CAD and its molecular relationships with other diseases.

## Introduction

Coronary artery disease (CAD) is the leading cause of morbidity and mortality worldwide and has a strong genetic basis [1]. Advances in genome-wide association studies (GWAS) have provided insights into lots of different genetic factors that contribute to the disease. Since 2007, the Wellcome Trust Case Control Consortium (WTCCC) and Framingham Heart Study have achieved duplicated validation of 9p21, and identification of 13 novel loci associated with CAD [2], [3]. More similar studies have also been performed in different populations, leading to the excavation of more CAD-related single nucleotide polymorphisms (SNPs) [4], [5]. As a result, population-based studies with thousands of patients and healthy controls included have identified more than 50 CAD-associated genetic loci in total [6], [7], [8], [9].

However, all these studies did not take into account the underlying genetic interplays or functional modules involved in CAD. Therefore, the genetic basis of CAD has remained largely unknown due to the limited understanding of just a small proportion of individual genetic variations. There is a developing consensus that genetic variations of CAD often function by sophisticated interactions through a modular fashion rather than individually [10]. With the improvement of computational methods, gene set-based association analysis (GSA) aimed at evaluating the joint effects of a defined gene set by quantifying the susceptibility or statistical significance of individual functional units, *e.g.*, pathways or biological processes, associated with clinical phenotypes [11].

In general, the gene sets in GSA can be generated according to the manually-curated pathways (*e.g.*, KEGG pathways), or gene lists related to specific functions. Based on the differences in the theoretical models used, the pathway-based methods can be classified into three categories, *i.e.*, functional enrichment analysis [12], [13], topology-based analysis [14], and multivariate statistical analysis. Hereinto, functional enrichment analysis is the most widely used method for the identification of enriched pathways related to the phenotype of interest due to its straightforward statistical model. For instance, Ghosh et al. [15] applied Reactome gene sets-based gene set enrichment analysis (GSEA) to reveal novel associations between key biological processes and CAD. By contrast, topology-based methods were proposed based on the fact that biological pathways are not simple lists of genes, but rather complex interactions between genes with specific topology. A list of topology-based methods and their applications have been reviewed previously [16]. However, so far, such methods are largely under development and their applications on CAD have rarely been reported. Instead, logistic kernel machine regression [17], [18], a semi-parametric regression model, is often used as the theoretical framework for multivariate analysis of multiple genetic variants in a gene set. It can be used to examine the significance of a gene set that corresponds to specific functional units such as pathways. By mapping the feature vector of genes or SNPs to a phenotype similarity space that indicates the similarity between people carrying these genes or SNPs, the logistic kernel machine regression analysis considers the linear interactions among the genes and thus shows advantage over the classical GSEA,

In this article, we report the findings from genome-wide and pathway-based analysis of a publicly-available GWAS dataset provided by WTCCC. We first systematically assessed the association of each KEGG pathway with CAD using the logistic kernel regression model. Then, we constructed an expanded genetic network by integrating gene–gene interactions involved in these susceptible pathways with their protein–protein interactions (PPIs). Finally, we identified the risk functional modules (subnetworks) for CAD by decomposing the built genetic network.

## Results

### SNP set test identified CAD susceptible KEGG pathways

The raw WTCCC genotyping data for CAD contained 482,247 SNPs from 5000 individuals (3000 controls and 2000 CAD patients). After data processing, 101,822 SNPs from 4864 individuals (2938 controls and 1926 CAD patients) were retained for further analysis. We defined the SNPs annotated to the genes of the same KEGG pathway as a SNP set. Hence, 276 pathway-based SNP sets were generated in total. These SNP sets were tested by logistic kernel machine regression model to evaluate whether they jointly (*i.e.*, pathway-based) contribute significantly to CAD risk. As a result, totally 6 pathways were identified to be significantly associated with CAD (Bonferroni-adjusted *P* < 0.05; Table 1). These include glycerolipid metabolism, glycosaminoglycan biosynthesis, and cardiac muscle contraction, as well as three disease-related pathways, *i.e.*, Alzheimer’s disease (AD), non-alcoholic fatty liver disease (NAFLD), and Huntington’s disease.

Interestingly, potential correlation between some of these pathways and CAD has been reported in previous studies. For example, de las Fuentes et al. [19] performed a pathway-based analysis of another independent GWAS dataset for the Framingham Heart Study and identified glycerolipid metabolism pathway to be significantly associated with CAD. In agreement with this study, a previous report showed that the level of serum triglyceride, a key element in glycerolipid metabolism, could be used as an effective predictor for CAD risk [20]. There is also evidence showing relevance of glycosaminoglycan biosynthesis with CAD. Glycosaminoglycan is present extensively in various cell types, to maintain the resilience and permeability of vascular wall or play a key role in anticoagulation and antihyperlipidemia [21]. Therefore, genetic variation in this pathway may cause dysfunction of blood vessels. It is not surprising that cardiac muscle contraction (hsa04260) was identified to be the risk pathway for CAD. Myocardial contraction is an initial process of potential shift of cardiac muscle cells, to produce longitudinal, radical, and rotational motion. Li et al. [22] applied an ultrasonic imaging technique and showed that cardiac muscle motion of CAD patients is significantly different from that of health subjects at various directions, implying the correlation between myocardial dysfunction and myocardial contraction in CAD patients.

Notably, our study revealed that three pathways related to other diseases were linked with CAD. It has been reported that among all kinds of fatty liver diseases examined, NAFLD shows the strongest correlation with CAD [23], and NAFLD patients have a higher risk for cardiovascular disease [24]. The correlation between AD, a chronic neurodegenerative disease, and CAD was also noticed because of occurrence of cognitive impairment in CAD patients [25]. Nevertheless, there is dearth of evidence supporting the relevance of CAD with Huntington’s disease, a Mendelian neurodegenerative disorder with autonomic dominant inheritance.

Taken together, most of the pathways identified in this study have clear evidence supporting their involvement in the underlying pathogenesis for CAD.

### CAD-related genetic network analysis identified *PIK3R1* and *APP* as hub genes

**Figure 1:**
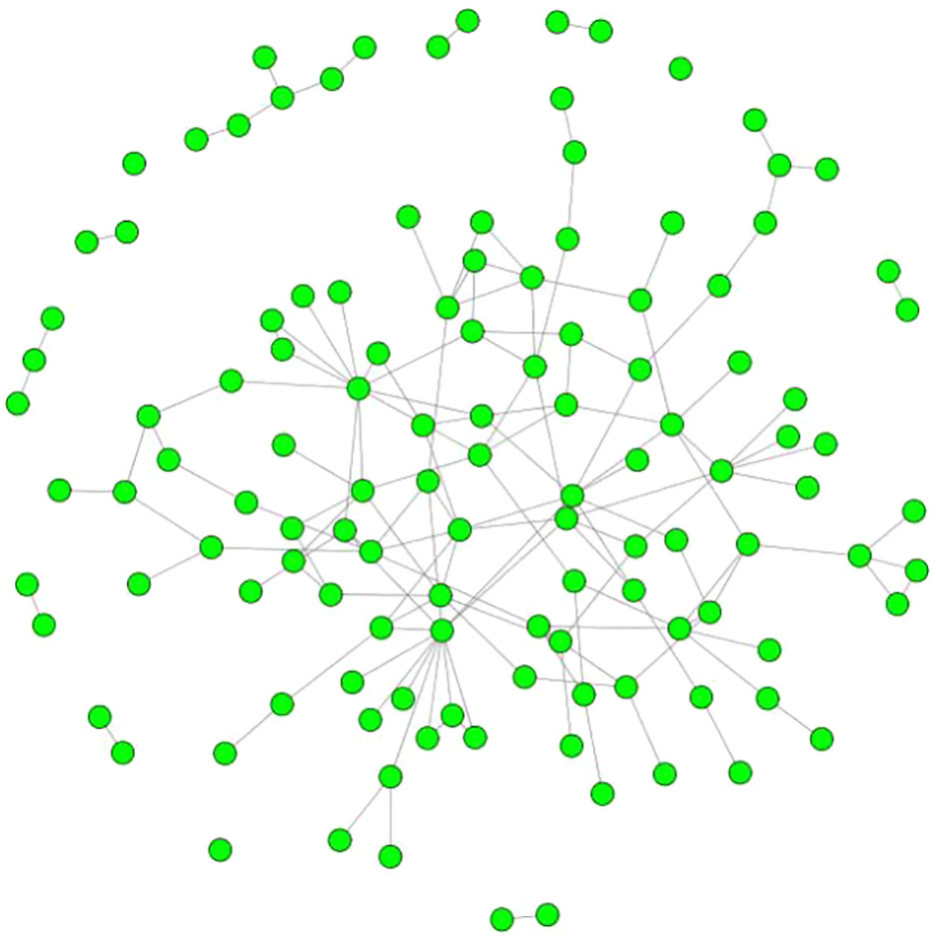
**Epistatic network for CAD** Epistasis analysis of all SNP–SNP pairs within or across the significant KEGG pathways was performed. Totally 186,640 SNP–SNP significant interactions (*P* < 0.05) were identified using PLINK. We then mapped the involved SNPs onto 121 genes and genetic network containing 149 gene–gene pairs was constructed by incorporating prior protein–protein interaction knowledge. Kolmogorov–Smirnov test showed that this network was a scale-free network with scaling exponent *α* = 3.0575 (*P* = 0.9345).

Epistasis analysis of all SNP−SNP pairs within or across the identified pathways was performed. Totally 186,640 SNP–SNP significant interactions (*P* < 0.05) were identified. We then mapped the involved SNPs onto genes. By integrating prior PPI knowledge, we constructed genetic networks using 121 unique genes and 149 gene−gene pairs. As shown in Figure 1, most of these genes were connected to each other, producing the largest sub-network with 95 unique genes and 135 edges. There were also 9 small sub-networks including seven sub-networks with only one edge, one sub-network with two edges, and one sub-network with three edges. Furthermore, there are three genes that were not linked to any other genes at all due to the lack of PPI evidence.

**Figure 2:**
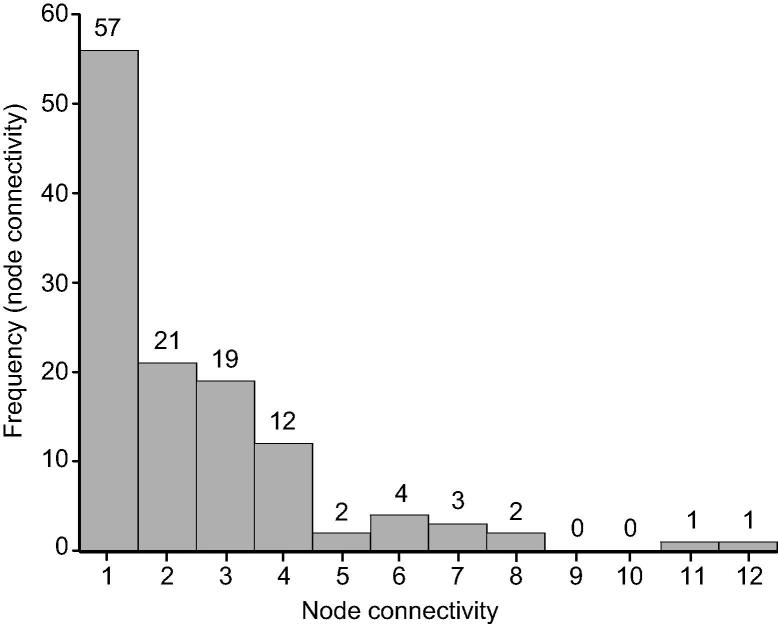
**Frequency distribution of node connectivity for the largest genetic network of CAD** Node distribution was analyzed for the largest subnetwork. Node connectivity was counted as the number of interacting genes according to significant SNP–SNP interactions. X axis indicates node connectivity and Y axis indicates the frequency of specific connectivity. The frequency of each degree is labeled on top of each bar.

We focused on the largest sub-network for the following topological analysis. The connection degree distribution of the largest sub-network (Figure 2) indicated that this network is not a random network. Furthermore, Kolmogorov–Smirnov (KS) test [26] showed that this network was a scale-free network with an exponential parameter *α* = 3.023 (*P* *=* 0.884). Among all the genes, two genes, *PIK3R1* and *APP*, showed the highest connectivity, which are connected with 11 (Bonferroni-adjusted *P* = 0.0041) and 12 other genes (Bonferroni-adjusted *P* *=* 0.00088), respectively. These two genes were thus defined as the hub genes.

*PIK3R1* encodes the regulatory subunit α of phosphatidylinositol 3-kinase (PIK3), which orchestrates a series of cell function regulation, such as cell proliferation, cell differentiation, apoptosis, and glucose transport [27]. PIK3 can be activated by angiotensin II, and the activated PIK3 plays a vital role in vascular smooth muscle cells through angiotensin II stimulated Ca^2+^ entry [28]. *APP*, which encodes *β*-amyloid precursor protein, is generally recognized to be closely related to AD [29]. Abnormal expression of *APP* can lead to dysfunction of endothelial cells due to cytotoxicity and damage induced by long-term exposure to A*β* peptide [30], [31]. These studies provide supporting evidence of *PIK3R1* and *APP* on the development of CAD.

### CAD-related genetic network module analysis reveals the involvement of various molecular functions

**Figure 3:**
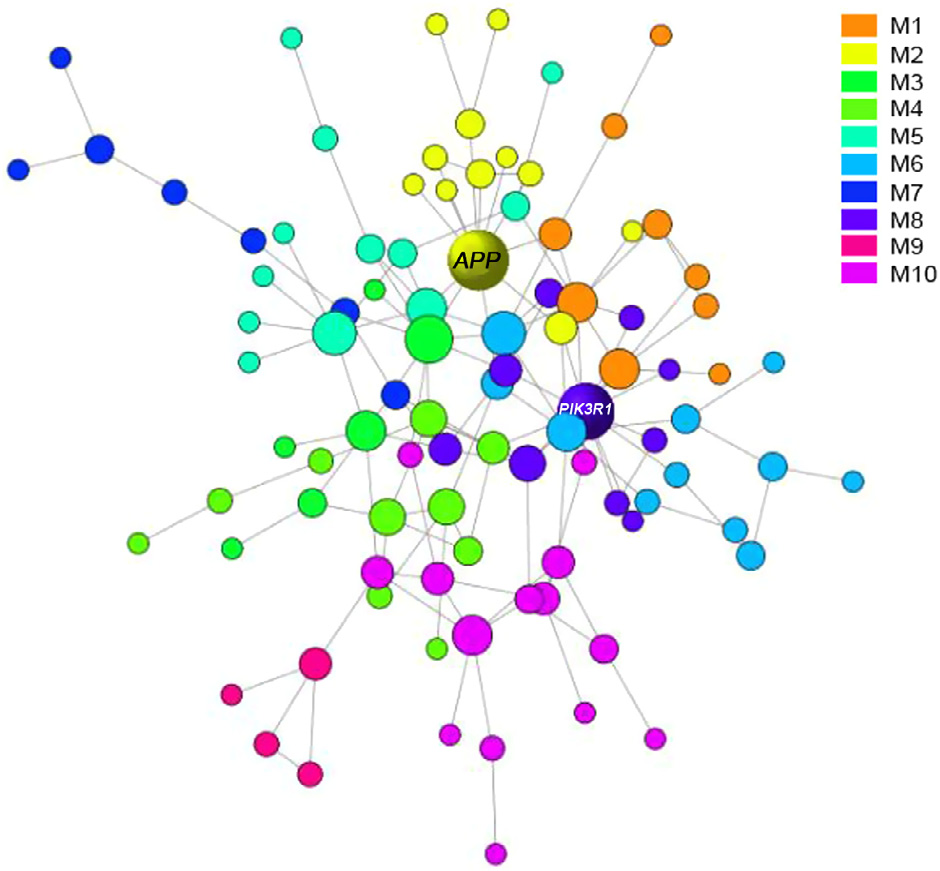
**Modular partitions of CAD risk genes** Risk modules of CAD were obtained by network decomposition with Newman algorithm. Gene nodes were sized by connectivity and partitioned to Modules 1–10. Hub genes *APP* and *PIK3R1* are labeled. Modules are color-coded and the detailed list of genes in each module is provided in Table S1.

To identify the most compact functional subnetworks, we further decomposed the largest network into smaller modular units. We obtained totally 10 modules, which consist of 4–14 genes (Figure 3). The corresponding gene lists can be found in Table S1. Interestingly, KS test revealed that all modules were scale-free with *P* values ranging 0.8–1.0, except the smallest one which only contained 4 genes (Table 2). The estimates of the scaling exponent (*α*) of power law distribution, KS testing statistics, and some other topological properties for each module are also shown in Table 2.

To understand the function involvements of each module, we performed a gene ontology (GO)-based enrichment analysis using the database for annotation, visualization and integrated discovery (DAVID). Only modules containing more than 10 genes were analyzed and the significantly-enriched GO terms for each module are listed in Table 3. We found that different modules had some characteristic functional involvements. For examples, M3 and M5 were significantly enriched with the cellular component of cytosol, M9 with cell nucleus, and M6 with neuron related structures, indicating that these modules had very different ‘working places’. In addition, these modules also exhibited varied molecular functions. For instances, M3 was linked to the lipid-related functions, M2 to peptidase activity, and M6 to cell adhesion molecule binding activity. Notably, M6 was also enriched with biological process neuronal activity, which could be the molecular bridge between AD and CAD.

Among all these enriched GO terms, phospholipase C activity (GO: 0004629) and cell adhesion molecule binding (GO: 0050839) took special attention. Phospholipase C (PLC), which is distributed widely in various tissues, is a key enzyme in phosphatidylinositol signaling pathway [31]. There exist different isozyme types of PLC, including PLC-*β*, PLC-*γ*, and PLC-*δ*. These isozymes possess conserved and specific domains, and therefore PLC activation may be induced in various ways [32]. PLC-*γ* is mostly activated through protein tyrosine kinases (PTKs). As a result, activated PLC-*γ* would induce a series of signal transduction, which may lead to trans-activation of epidermal growth factor (EGF), a key element in inhibition of vascular wall deposition and thrombogenesis [33], [34]. Additionally, by mediating inter-cellular interactions, cell adhesion molecules (CAMs) can regulate multiple biological processes, such as signal transduction, inflammation and immune responses, coagulation and tissue repairing [35]. The changes in adhesion of vascular endothelial cell surface, *e.g.*, caused by injury, promote monocyte adhesion. During the migration and transformation to macrophages, monocyte may stimulate lymphocytes to produce multiple cytokines, thus promoting proliferation of smooth muscle and formation of fibrous plaque eventually [36].

## Discussion

Studies of genetic variants in various molecular biological processes will greatly promote our understanding of CAD and its pathogenesis. In the present study, a pathway-based approach is applied to identify the risk pathways and functional modules for CAD. This study demonstrated that multiple pathways may be involved in the underlying molecular processes for CAD, such as cardiac muscle contraction, glycerolipid metabolism, and glycosaminoglycan biosynthesis. Meanwhile, this study reveals that some other diseases, *e.g.*, non-alcoholic fatty liver disease, Huntington’s disease, and AD, may share molecular mechanisms with CAD. Furthermore, 2 hub genes, *PIK3R1* and *APP*, and 6 risk functional modules for CAD have been identified. Our findings are different from Liu’s study [37]. Using gene co-expression network analysis, Liu et al. identified specific modules and hub genes that are mainly related to membrane-associated processes and hypertrophic cardiomyopathy pathway. The possible reasons for the difference may be manifold, one of which is the difference in the choice of omics data type. They evaluated gene expression changes at mRNA level, whereas we analyzed the genomic mutations within DNA sequences. Overall, our study provides new insights into the molecular basis for CAD and its molecular relationships with several related diseases.

Genetic studies are traditionally based on single gene analysis, which poses tremendous challenges for elucidating complicated genetic interplays involved in complex human diseases. Modern pathway-based analysis allows a comprehensive understanding of the molecular mechanisms underlying complex diseases by considering the joint effect and integrality as function unit of multiple genes. Extensive studies utilizing pathway-based analysis have significantly advanced our capacity to explore large-scale omics data that have been rapidly accumulating in biomedical fields [38].

Pathway-based approach has some advantages over the conventional GWAS approach. The singe-locus analysis widely used in GWAS is only capable of capturing a small portion of susceptible SNPs with prominent marginal effects, leaving the important genetic component, such as epistasis or joint effects between multiple genes, undetected. Identifying the complex interplays among multiple genes in the genome-wide context is an essential task to systematically unravel the molecular mechanisms underlying complex diseases. In this study, we employed the newly-developed logistic kernel machine regression model in the pathway-level analysis to capture the joint effects of multiple genes involved in the pathways. In this way, we are not only able to avoid the curse of ‘high dimensionality and small sample size’ associated with analysis of GWAS data, but also able to estimate the missing genetic components and epistasis, thus helping elucidate the sophisticated molecular interplays between or across the risk pathways for CAD.

There are also limitations in this study. First, the whole pathogenic process of CAD may involve a long cascade of multiple biological pathways. Ideally, the accumulative effects of these risk pathways on CAD should be examined, which is practically difficult to achieve because of the unpredictability of joint action among different pathways and the possibility of over-fitting. Second, we replaced missing genotypes with the most frequent allele. Such simplification for data imputation could lead to expansion of major alleles, resulting in a decreased minor allele frequency (MAF). On the other hand, genotype imputation is a complex process in GWAS research, which can address the failures occurring during genotyping assay to some extent [39], [40]. However, in practice, most imputation methods require external reference panel of SNPs that may introduce noise of genetic background, and the success of imputation is largely determined by the patterns of linkage disequilibrium (LD) [41]. In our analysis, the missing genotypes were imputed after filtering SNPs and subjects with missing rate ⩾5%, which help restrain the expansion of major alleles. Moreover, a gene-set-based method rather than a single-locus-based method was applied in our further analysis, reducing the bias of single SNPs resulting from the imputation. Third, in the epistatic analysis, we used the nominal *P* value of 0.05 to identify the putative epistatic gene pairs, which could lead to an inflated type I error. Correction for multiple tests is a very complicated and challenging issue for the analysis of large-scale GWAS data, especially for epistatic analysis because of correlation of gene pairs or correlations stemming from LD. To address this concern, we applied an additional criterion of experimentally-confirmed PPI support for gene pairs, which might alleviate the issue of the inflated type I errors to some extent. Finally, this study only integrated GWAS data with PPI data for genetic network analysis. Integrating more omics data such as epigenetic or epidemiological data would help illustrate the genetic, epigenetic, and environmental factors for CAD, which is the focus in our future studies.

## Materials and methods

### Data sources

WTCCC genotyping data of 482,247 SNPs from 2000 CAD subjects and 3000 health control subjects [42] were analyzed in this study. For identification of susceptible pathways, 283 human pathways [43], [44] were extracted and downloaded from KEGG database. We removed the more general pathways that contain several specific pathways, and ultimately included 276 KEGG pathways. Genetic information for mapping SNPs to genes was extracted from Ensembl/GRCh37 [16]. Information on PPIs was retrieved from the Human Protein Reference Database (HPRD) [45] for genetic networking (epistatic interactions). To enhance the reliability of the depicted genetic relationships, only experimentally-confirmed PPIs were taken as the prior knowledge.

### Data preprocessing

To improve the data quality, several data preprocessing procedures were performed. First, SNPs were excluded if they did not meet all the following criteria: (1) genotype missing rate < 5%, (2) individual missing rate < 5%, and (3) MAF > 0.01. Furthermore, all the included loci must meet Hardy–Weinberg equilibrium (HWE) proportions (*P* > 1 × 10^−4^) in the control group. Second, for loci with missing values after the filtering above, missing genotypes were replaced with the most frequent one. Third, to remove data redundancy due to LD, only tag SNPs that were representative in the corresponding genomic regions were utilized for the current analysis. For identification of these tag SNPs, each individual chromosome was scanned using the ‘moving window’ method in which the window size was set to 50 SNPs with step length of 5 SNPs. The cutoff of LD *r*^2^ was set as 0.8. Finally, SNPs were considered as mapped onto genes if these SNPs are situated in the flanking regions spanning from 5 kb upstream to 5 kb downstream of the genes, as described previously [46]. All the data processing procedures were performed using PLINK program [47] and R platform (http://www.r-project.org/).

### Identification of CAD susceptible pathways

Logistic kernel machine regression model was applied to identify the susceptible pathways related to CAD. Suppose that a pathway contains *p* SNPs (their genotypes are denoted as $z_{i*}$) and $P(y_{i}=1)$ be the probability of the *i*th subject being affected (*i.e.*, who has the disorder). This model can be described as follows:

(1) $$\text{logit}P(y_{i}=1)=\alpha_{0}+h(z_{i1}\text{,}z_{i2}\text{,}\cdots \text{,}z_{\mathrm{ip}})$$

where $\alpha_{0}$ is the intercept, and h(·) is a general function of *p* SNPs contained in the pathway, which is often defined as a positive, semi-definite kernel function K(·,·). In this study, this kernel function is defined as

(2) $$K(Z_{i}\text{,}Z_{i^{\prime }})=\overset{p}{\underset{j=1}{\sum }}w_{j}z_{\mathrm{ij}}z_{i^{\prime }j}$$

where the weight $w_{i}$ is calculated as described previously [48]. More intuitively, *K*(.,.) can be viewed as a function that measures the similarity between two individuals based on the genotypes of the SNPs in the SNP set. There are three options for *K*(.,.): the linear, Gaussian, and identical-by-state kernels. The null hypothesis for testing a pathway is that its overall effect is zero, *i.e.*, *H*_0_: h(·) = 0. The significance of the pathway-based SNP set was tested by *Q* statistics that follows a $\chi^{2}$ mixed distribution. To adjust for multiple pathways to be evaluated, Bonferroni correction was applied and significance was claimed if *P* × *N* < 0.05 (*N* is the number of pathways evaluated). More details about the methods used are described previously [48], [49].

### Genetic networking

To further elucidate the underlying interplays between multiple genetic variants within a pathway or across different pathways, an expanded genetic network was constructed by integrating statistical gene−gene interactions involved in the identified susceptible pathways with their PPI knowledge. First, a pairwise epistatic analysis was performed using PLINK for the SNPs that were annotated to these susceptible pathways. To avoid possible loss of some meaningful interactions, all SNP pairs with *P* < 0.05 were retained and then translated into putative gene–gene interactions. However, the gene pairs that were finally used to construct the gene network must have support from the HPRD PPI knowledge database. After the gene network was built, its topological properties (*e.g.*, connectivity, betweenness, and cluster coefficient) were examined. Network hub genes were identified by testing whether the connectivity of a certain gene node was equal to or greater than that expected based on a Poisson distribution [25]. Finally, to analyze the network modularity, the Newman algorithm [50] was used to decompose the network into the most compact modules. These putative modules for CAD were further investigated in terms of their topological properties and functional involvements. All the aforementioned network analysis and visualization were performed in R/igraph package.

### Function enrichment analysis

To characterize the functional involvement of the putative modules for CAD, GO analysis was performed for each module using DAVID [51], [52] with the whole human genome genes as background, and the gene list within each module as foreground. To control false positive rate of significance of GO terms, Bonferroni correction was used. In order to better characterize the putative modules, we reported the GO terms with node depth ⩾4. The information for GO hierarchy was retrieved from Bioconductor GO.db and the node depth for each GO term was defined by the minimum distance between target GO term and GO root term in the tree structure.

## Authors’ contributions

SQR conceived the project and wrote the manuscript. XZ and YZL performed the analysis and wrote the manuscript. XZ, YDC, JQ, LJ, YT, ML, NZ, YL participated in writing the computing codes and analyzing the public datasets. All authors read and approved the final manuscript.

## Competing interests

The authors declare no competing interests.

## Figures and Tables

**Table 1:** Significant susceptible pathways for CAD

**Pathway ID**	**Pathway name**	***P* value**	**Adjusted *P* value**
hsa00561	Glycerolipid metabolism	7.57E−08	2.09E−05
hsa00532	Glycosaminoglycan biosynthesis	3.92E−13	1.08E−10
hsa05010	Alzheimer’s disease	3.59E−05	9.92E−03
hsa04932	Non-alcoholic fatty liver disease	1.60E−04	4.44E−02
hsa05016	Huntington’s disease	2.31E−05	6.37E−03
hsa04260	Cardiac muscle contraction	7.22E−05	1.99E−02

*Note*: Logistic kernel machine regression analysis was performed on SNP sets annotated to the same pathways for evaluation of significant KEGG pathways (*P* < 0.05). To calculate adjusted *P* values, Bonferroni’s adjustment was conducted for the number of KEGG pathways evaluated. CAD, coronary artery disease.

**Table 2:** The topological features of the risk modules for CAD

**Module**	**No. of nodes**	**No. of edges**	**Network diameter**	**Scaling exponent**	**KS statistic D**	***P* value**
M1	9	10	5	3.051	0.1107	1
M2	12	13	4	2.421	0.0977	1
M3	10	12	6	2.801	0.1288	0.9994
M4	6	5	4	2.016	0.1763	0.9922
M5	12	12	6	2.560	0.0951	1
M6	11	12	5	2.965	0.1598	0.9868
M7	7	6	5	1.988	0.2286	0.8577
M8	10	12	3	3.139	0.0936	1
M9	14	16	5	1.868	0.1560	0.8849
M10	4	4	2	–	–	–

*Note*: CAD, coronary artery disease; KS, Kolmogorov–Smirnov.

**Table 3:** The GO terms enriched for each risk module for CAD

**Module**	**GO ID**	**Category**	**GO term**	**Depth of GO hierarchy**	***P* value**
M2	GO:0008233	MF	Peptidase activity	4	2.08E−03

M3	GO:0005829	CC	Cytosol	5	6.78E−03
	GO:0004629	MF	Phospholipase C activity	7	1.62E−02
	GO:0003707	MF	Steroid hormone receptor activity	4	4.02E−02

M5	GO:0031264	CC	Death-inducing signaling complex	4	5.98E−04
	GO:0005741	CC	Mitochondrial outer membrane	5	4.75E−03
	GO:0005829	CC	Cytosol	5	3.20E−02

M6	GO:0007612	BP	Learning	6	4.25E−03
	GO:0035235	BP	Ionotropic glutamate receptor signaling pathway	7	7.69E−03
	GO:0060079	BP	Regulation of excitatory postsynaptic membrane potential	6	3.59E−02
	GO:0048169	BP	Regulation of long-term neuronal synaptic plasticity	6	4.38E−02
	GO:0030426	CC	Growth cone	4	7.37E−04
	GO:0008328	CC	Ionotropic glutamate receptor complex	4	4.38E−03
	GO:0030425	CC	Dendrite	5	1.99E−02
	GO:0050839	MF	Cell adhesion molecule binding	4	2.16E−02

M8	GO:0005942	CC	Phosphoinositide 3-kinase complex	4	1.82E−03

M9	GO:0044451	CC	Nucleoplasm part	5	7.55E−05
	GO:0005667	CC	Transcription factor complex	4	8.80E−04
	GO:0000790	CC	Nuclear chromatin	6	4.99E−02

*Note*: CAD, coronary artery disease; MF, molecular function; CC, cellular component; BP, biological process. To count the depth of GO terms, the depth of the root term in each category was taken as 1.

## Supplementary material

Supplementary Table S1 Genes contained in each risk module of CAD.
