# Supplementary material for: Identification of Risk Pathways and Functional Modules for Coronary Artery Disease Based on Genome-wide SNP Data
Source: Genomics Proteomics Bioinformatics. 2016 Dec 11;14(6):349–56. doi: 10.1016/j.gpb.2016.04.008 (PMC5200919; doi:10.1016/j.gpb.2016.04.008)
Supplement: Supplementary Table S1 — Genes contained in each risk module of CAD. [file mmc1.docx]

**Table S1** **Genes contained in each risk module of CAD**

| **Module** | **Genes** |
| --- | --- |
| M1 | *INSR*, *GRM5*, *SNCA*, *CALM1*, *COX4I2*, *IK3R3*, *CYCS*, *CALM2*, *CALM3* |
| M2 | *LRP1*, *APBB1*, *BACE2*, *BACE1*, *NCSTN*, *LPL*, *NAE1*, *LIPC*, *APH1A*, *PSEN2*, *APP*, *PSEN1* |
| M3 | *MAPK8*, *PPARA*, *RXRA*, *PPARG*, *PPARGC1A*, *GNAQ*, *PLCB1*, *MAPK1*, *NRF1*, *PLCB2* |
| M4 | *CASP3*, *TGM2*, *HAP1*, *HTT*, *TBP*, *DCTN1* |
| M5 | *CASP8*, *HIP1*, *BAX*, *FAS*, *BID*, *APAF1*, *CASP9*, *CLTA*, *SLC25A4*, *CLTC*, *IFT57*, *FADD* |
| M6 | *CAPN2*, *GSK3B*, *CAPN1*, *MAPT*, *ACTC1*, *GRIN2B*, *PIK3CA*, *GRIN2A*, *CDK5*, *CDK5R1*, *DLG4* |
| M7 | *PPP3CA*, *ERN1*, *TNFRSF1A*, *MAP3K5*, *BAD*, *TRAF2*, *PPP3R1* |
| M8 | *PIK3R1*, *CASP7*, *PIK3CB*, *AKT1*, *PIK3CD*, *RAC1*, *IRS1*, *CDC42*, *MME*, *FASLG* |
| M9 | *RELA*, *TP53*, *MAPK10*, *SIN3A*, *JUN*, *MAPK9*, *CREBBP*, *CREB5*, *EP300*, *BCL2L11*, *HDAC2*, *SP1*, *RCOR1*, *VDAC1* |
|  |  |
| M10 | *POLR2A*, *ITCH*, *POLR2C*, *POLR2F* |
